# Strain pattern and T‐wave alterations are predictors of mortality and poor neurologic outcome following stroke

**DOI:** 10.1002/clc.23348

**Published:** 2020-02-22

**Authors:** Gabriel P. Braga, Renato S. Gonçalves, Marcos F. Minicucci, Rodrigo Bazan, Leonardo A. M. Zornoff

**Affiliations:** ^1^ Botucatu Medical School, Neurology Department São Paulo State University (Unesp) Botucatu Brazil; ^2^ Botucatu Medical School, Internal Medicine Department São Paulo State University (Unesp) Botucatu Brazil

**Keywords:** electrocardiogram, mortality, neurologic disability, outcomes, prediction, stroke

## Abstract

**Background:**

Stroke is associated with electrocardiogram (ECG) abnormalities. However, the role of strain pattern as predictor of poor neurologic outcome and mortality after stroke has not yet been demonstrated.

**Hypothesis:**

ECG abnormalities, with a particular focus on ST‐segment changes, are predictors of mortality and neurologic disability 90 days after stroke.

**Methods:**

Patients with up to 24 hours of stroke were prospectively recruited. An ECG was taken at the time of admission. The patients' clinical evolution was evaluated during hospitalization and after discharge by means of a prescheduled return in 90 days. The degree of disability was measured by the modified Rankin scale (mRs). In relation to the mRs, patients were divided into those with scores from 0 to 2 and those with scores equal to or greater than 3 at the end of the observation period.

**Results:**

Of the 112 patients studied, 29 (25.8%) died during the study period. Patients who died presented higher National Institute of Health Stroke Scale and mRs scores on admission, elevated biomarkers of myocardial necrosis, and abnormalities on the ECG. The prevalence of ECG abnormalities was 63%. A logistic regression model showed that strain pattern and T‐wave alterations were predictors of mortality (odds ratio [OR]: 12.970, 95% confidence interval [CI]: 1.519‐110.723, *P* = .019; OR: 3.873, 95% CI: 1.135‐13.215, *P* = .031, respectively) and mRs at 90 days (OR: 12.557, 95% CI: 1.671‐94.374, *P* = .014; OR: 15.970, 95% CI: 3.671‐69.479, *P* < .001, respectively) after stroke, adjusted by sex, age, stroke subtype, entrance NIH, previous mRs score, and stroke thrombolysis.

**Conclusion:**

Strain pattern and T‐wave alterations were predictors of mortality and poor neurologic outcome 90 days after stroke.

## 1 INTRODUCTION

Stroke is a major cause of morbidity and mortality around the world. In addition to representing a profound burden of physical and psychological suffering for patients and their families, this pathology also represents enormous social costs, whether measured in days of lost work or the costs of treatment, secondary prevention, and rehabilitation.^1^

A relevant challenge in the management of stroke is that this pathology is associated with heterogeneous prognoses. Therefore, predictors of poor outcome after an acute stroke, including age, severity of stroke, clinical subtype, previous stroke history, score on the Glasgow Coma Scale, arm strength, ability to walk, and prestroke dependence, are critical for the best management of patients.^1^ In recent years, particular attention has focused on the electrocardiogram (ECG).

It is well known that stroke can induce ECG abnormalities and it has been hypothesized that these alterations are associated with adrenergic hyperstimulation, with consequent myocyte injury.^2^, ^3^, ^4^

Several^5^, ^6^, ^7^, ^8^, ^9^, ^10^, ^11^ but not all^12^ studies suggest that ECG abnormalities, including alterations in T waves, prolongation of the QT interval, and ST changes, can be predictors of mortality after stroke. However, the kind of ST‐segment abnormality associated with poststroke prognosis remains to be determined. Likewise, it is unknown whether ECG abnormalities can predict a poor neurologic outcome.

Therefore, the main objective of our study was to analyze ECG abnormalities, with a particular focus on ST‐segment changes as predictors of mortality and neurologic disability 90 days after stroke. In addition, we assessed associations between ECG abnormalities and biomarkers of myocardial necrosis and clinical stroke syndromes.

## 2 METHODS

All procedures were approved by the ethics committee of our institution (CEP 3827‐2011) and all participants provided their written consent. From March 2012 to March 2013, consecutive patients with up to 24 hours of stroke were prospectively recruited.

Acute stroke was diagnosed in the presence of a sudden episode of focal dysfunction of the brain or retina and/or evidence of focal infarction or hemorrhage on cranial computed tomography (CT).^1^ Exclusion criteria were as follows: nonvascular acute neurologic deficit; active malignancy; infection; end‐stage cardiac, pulmonary, or hepatic disease; pregnancy; age less than 18 years; previous myocardial infarction; and serious valve disease.

At admission, data on patient characteristics were recorded, including waist circumference, body mass index, age, gender, heart rate, cardiovascular risk factors, concomitant diseases, medical and neurological complications, medical treatment, and data regarding clinical presentation and prehospital delay.

A CT scan was performed without contrast on Shimadzu SCT‐7000 tomograph with infratentorial sections of 3‐mm thickness and 5‐mm spacing, 10‐mm thickness supratentorial spacing and 10‐mm spacing for the acquisition of images through the supraorbitomeatal angulation of the apparatus. The examination was performed at admission and tomographic control at 48 to 72 hours after admission.

An ECG was performed at the time of admission with an eight‐channel Eletrotouch EP‐3 Philips Dixtal with 12 leads, a 50/60 Hz filter, N gain, and 25 mm/s velocity in automatic mode. It was evaluated by a cardiologist who had no clinical information about the patients. The following predictive variables were analyzed: rhythm (sinus or atrial fibrillation [AF]), heart rate (in beat per minute), duration of QRS measured in derivation V3 (in milliseconds), intraventricular conduction block, pathologic Q wave (S‐score) in all leads (in millimeters), an abnormal ST‐segment, T‐wave abnormality, QT interval corrected by the Bazzet formula (in milliseconds), and extrasystoles. ST‐segment abnormalities were assessed by any of the following conditions: supra‐ or nondistal with respect to point J, secondary repolarization abnormalities, and strain pattern (descending ST‐segment depression over 0.5 mm, with negative and asymmetric T wave).

The biomarkers of myocardial necrosis that were evaluated included total plasma creatine kinase (CPK), MB‐fraction (CPK‐MB), and troponin I. The CPK dosage was performed using the dry chemistry method; the enzymatic CPK‐MB was performed on the Johnson & Johnson Fusion 5.1 apparatus with a sample taken on the patient's arrival at the emergency room and was considered normal for values less than16 U/L. Troponin I was measured with the BioMériux Lives device, also collected at the patient's arrival, at least 6 hours after the onset of neurologic deficit. It was considered normal for values less than 0.01 μg/L.

The patients' clinical evolution was evaluated during hospitalization and after discharge by means of a prescheduled return in 90 days.

The degree of disability was measured by the modified Rankin scale (mRs)^13^ dichotomized according to the prognostic definition adopted in studies on the thrombolytic treatment of stroke.^14^ At 90 days, a poor prognosis is indicated by a Rankin score between 3 and 6. For patients who, on admission to the emergency room, are already in coma and on mechanical ventilation, the maximum score was taken on the NIHSS (National Institute of Health Stroke Scale).^15^

Medical complications were considered to include acute coronary syndrome, sudden death, AF or flutter, other arrhythmias, hypertensive crisis, acute heart failure, acute pulmonary edema, pulmonary thromboembolism (cardiac complications), pneumonia, urinary tract infection, deep venous thrombosis, gastrointestinal hemorrhage, and acute renal failure.^16^, ^17^, ^18^, ^19^, ^20^, ^21^, ^22^, ^23^, ^24^, ^25^

### 2.1 Statistical analysis

Data are expressed as the mean ± SD, the median (including the lower and upper quartiles) or as percentages. Comparisons between two groups for continuous variables were performed using Student's *t* test or the Mann‐Whitney test. Comparisons between two groups for categorical variables were performed using the chi‐square test or Fisher's exact test. A simple logistic regression model was used to predict outcomes. Variables were adjusted with parameters that indicated a significant difference in the univariate analysis or for factors that influenced the outcomes analyzed. The only exceptions were variables with high collinearity. Data analysis was performed using the SigmaPlot software for Windows v12.0 (Systat Software Inc., San Jose, California). *P* values lower than .05 were considered statistically significant.

## 3 RESULTS

A total of 145 patients were evaluated. Of these, 18 patients presented time to onset of symptoms greater than 24 hours of evolution or stroke recurrence and were excluded. During follow‐up, six patients who were diagnosed with other clinical conditions that mimicked stroke were excluded, as were five other patients with unreadable ECGs or with error in the registry. In addition, four patients withdrew consent for participation in the study, leaving a sample of 112 patients.

Complications during hospitalization were observed in 24 (21.4%) patients. A simple logistic regression model indicated that when hospitalization was complicated, there was approximately an 11‐fold greater chance of a poor neurologic outcome (odds ratio [OR]: 11.1, 95% confidence interval [CI]: 3.08‐40.13, *P* < .001). Cardiac complications were observed in 19 (16.4%) patients; their negative influence on the neurologic prognosis was stronger than that of the medical complications (OR: 12.3, 95% CI: 2.68‐56.4, *P* < .001).

In relation to the mRs, patients were divided into those with scores from 0 to 2 and those with scores equal to or greater than 3 at the end of the observation period. The demographic and clinical data are shown in the Table 1. Patients with higher mRs scores at 90 days presented higher NIHSS and mRs at admission, elevated biomarkers of myocardial necrosis, and abnormalities on the ECG in comparison with patients with lower mRs at 90 days.

**Table 1:** Demographic and clinical data on 112 patients with stroke

	90‐day mortality			90‐day RANK		
Variables	Yes (n = 29)	No (n = 83)	*P* value	0‐2 (n = 57)	3‐6 (n = 55)	*P* value
Age, (years)	75.0 (60.0‐85.0)	67.0 (60.0‐79.0)	.07	67.0 (60.0‐78.5)	72.0 (60.0‐83.0)	.09
Male, n (%)	14 (48.3)	44 (53.0)	.82	31 (54.4)	27 (49.1)	.71
Stroke subtype			.62			.39
Ischemic stroke	23 (79.3)	71 (85.5)		50 (87.7)	44 (80.0)	
Others	6 (20.7)	12 (14.5)		7 (12.3)	11(20.0)	
Entrance NIHSS	17.5 (7.3‐23.8)	4.0 (2.0‐10.3)	<.001	3.0 (2.0‐7.0)	13.0 (5.0‐22.0)	<.001
Previous mRs	0 (0‐2)	0 (0‐0)	.01	0 (0‐0)	0 (0–2)	<.001
Hypertension, n (%)	22 (75.9)	60 (72.3)	.95	42 (73.7)	40 (72.7)	.91
Diabetes, n (%)	6 (20.7)	17 (20.5)	.82	11 (19.3)	12 (21.8)	.92
Smokers, n (%)	7 (24.1)	30 (36.1)	.30	20 (35.1)	17 (30.9)	.79
Atrial fibrillation, n (%)	7 (24.1)	10 (12.0)	.23	6 (10.5)	11 (20.0)	.26
CAD, n (%)	2 (6.9)	10 (12.0)	.65	5 (8.8)	7 (12.7)	.71
CK, (U/L)	87.0 (49.5‐178.5)	74.0 (47.0‐124.0)	.30	70.5 (40.3‐120.0)	83.0 (54.0‐180.0)	.16
CK‐MB, (U/L)	23.0 (16.0‐34.5)	17.0 (11.0‐25.0)	.009	16.0 (10.0‐23.0)	21.5 (14.5‐31.8)	.004
Troponin, (μg/L)	0.01 (0.01‐0.06)	0.01 (0.01‐0.01)	.001	0.01 (0.01–0.01)	0.01 (0.01‐0.01)	.03
ECG abnormalities, n (%)	21 (72.4)	40 (48.2)	.03	22 (38.6)	39 (70.9)	.001
Stroke thrombolysis, n (%)	7 (24.1)	7 (8.4)	.06	7 (12.3)	7 (12.7)	.83

*Note:* Data are expressed as the median (including the lower and upper quartiles) or percentages.

Abbreviations: CAD, coronary artery disease; mRs, modified Rankin scale; NIHSS, National Institute of Health Stroke Scale.

The prevalence of ECG abnormalities was 63%. The more frequent alterations were T‐wave alterations (36%), QTc prolongation (26%), AF (25%), and ST‐segment changes (23%). In total, 50% of patients with hemorrhagic stroke presented with QTc prolongation. Data showing which ECG abnormalities were associated with a poor prognosis after stroke are presented in Table 2. AF and a prolonged QTc were associated with a greater likelihood of mortality. However, only strain pattern and T‐wave alterations were associated with both mortality and mRs at 90 days.

**Table 2:** Relation between ECG abnormalities and outcomes at 90 days

	90‐day mortality			90‐day RANK		
Variables	Yes (n = 29)	No (n = 83)	*P* value	0‐2 (n = 57)	3‐6 (n = 55)	*P* value
Atrial fibrillation	11 (37.9)	13 (15.7)	.02	8 (14.0)	16 (29.1)	.09
Heart rate	92 (70‐120)	78 (66‐92)	.03	80 (68‐99)	80 (67‐100)	.94
Intraventricular block	7 (24.1)	13 (15.7)	.38	7 (12.3)	13 (23.6)	.17
Pathologic Q wave	2 (6.9)	4 (4.8)	.96	2 (3.5)	4 (7.3)	.71
ST abnormalities	14 (48.3)	11 (13.3)	<.001	7 (12.3)	18 (32.7)	.015
Strain	8 (27.6)	4 (4.8)	.002	2 (3.5)	10 (18.2)	.025
Repolarization abnormalities	3 (10.3)	5 (6.0)	.68	3 (5.3)	5 (9.1)	.66
T abnormalities	14 (48.3)	24 (28.9)	.049	12 (21.1)	26 (47.3)	.005
QTc	11 (37.9)	11 (13.3)	.006	7 (12.3)	15 (27.3)	.06

*Note:* Data are expressed as the median (including the lower and upper quartiles) or percentages.

Of the 112 patients studied, 29 (25.8%) died during the study period. Cardiac complications correlated with a greater chance of evolution to a fatal outcome than general medical complications (OR: 10.4, 95% CI: 3.5‐31.5, *P* < .001; vs OR: 6.8, 95% CI: 2.5‐18.2, *P* < .001).

Mortality data are shown in Table 1. Patients who died presented higher NIHSS and mRs scores on admission, elevated biomarkers of myocardial necrosis, and abnormalities on the ECG in comparison with patients who survived.

A logistic regression model showed that strain pattern and T‐wave alterations were predictors of mortality and mRs (Table 3) at 90 days after stroke in both unadjusted and adjusted analyses.

**Table 3:** Logistic regression model for the prediction of 90‐day mortality and Rankin score 90 days after stroke in 112 patients

Outcome	Variable	OR	95% CI	*P* value
90‐day mortality	Strain^a^	2.067	2.160‐28.890	.002
	Strain^b^	14.185	1.506‐133.612	.020
	Strain^c^	12.970	1.519‐110.723	.019
	T‐wave abnormality^a^	2.722	1.099‐6.745	.031
	T‐wave abnormality^b^	3.952	1.035‐15.088	.044
	T‐wave abnormality^c^	3.873	1.135‐13.215	.031
Rankin score 90 days after stroke	Strain^a^	6.250	1.301‐30.014	.022
	Strain^b^	20.134	1.852‐218.859	.014
	Strain^c^	12.557	1.671‐94.374	.014
	T‐wave abnormality^a^	3.500	1.510‐8.114	.004
	T‐wave abnormality^b^	11.936	3.011‐47.310	<.001
	T‐wave abnormality^c^	15.970	3.671‐69.479	<.001

a Unadjusted.

b Adjusted by entrance NIHSS (National Institute of Health Stroke Scale), previous modified Rankin scale score, and troponin.

c Adjusted by sex, age, stroke subtype, entrance NIHSS, previous modified Rankin scale score, and stroke thrombolysis.

There was no association between ECG abnormalities (Table 4), strain pattern, and T‐wave alterations (data not showed) with biomarkers of myocardial necrosis and clinical stroke syndromes as assessed by the Oxfordshire Community Stroke Project (Bamford) classification.

**Table 4:** Association between ECG abnormalities with biomarkers of myocardial necrosis and clinical stroke syndromes

	ECG abnormalities		
Variables	Yes (n = 61)	No (n = 51)	*P* value
CPK, (U/L)	82.0 (48.3‐180.5)	73.0 (47.0‐126.5)	.43
CPK‐MB, (U/L)	20.0 (12.8‐28.0)	16.0 (10.5‐25.0)	.11
Troponin, (ng/mL)	0.01 (0.01–0.01)	0.01 (0.01‐0.01)	.10
Bamford classification, n (%)			.58
LACS	11 (18.0)	14 (27.4)	
PACS	28 (45.9)	20 (39.2)	
POCS	9 (14.8)	5 (9.8)	
TACS	8 (13.1)	6 (11.8)	

*Note:* Data are expressed as the median (including the lower and upper quartiles) or percentages.

Abbreviations: LACS, lacunar syndrome; PACS, partial anterior circulation syndrome; POCS, posterior circulation syndrome; TACS, total anterior circulation syndrome.

## 4 DISCUSSION

The objective of our study was to analyze ECG abnormalities with a particular focus on ST‐segment changes as predictors of mortality and neurologic disability 90 days after stroke. In addition, we assessed the association between ECG abnormalities with biomarkers of myocardial necrosis and clinical stroke syndromes. Our data show that both strain pattern and T‐wave alterations were predictors of both mortality and a poor neurologic outcome 90 days after stroke. Importantly, there was no association between ECG abnormalities and biomarkers of myocardial necrosis and clinical stroke syndromes.

The role of ECG after stroke has been widely analyzed in recent years, indicating that ECG alterations are commonly seen after brain injury even without preexisting heart disease, ranging from 30% to 90% depending on the study population.^26^, ^27^ The incidence of these manifestations is higher in patients with subarachnoid hemorrhage than in those with ischemic stroke.^4^ The commonly seen ECG changes include T‐wave inversion, U‐wave abnormalities, abnormalities of the ST segment, and prolongation of the QT interval. Therefore, the types of ECG alterations found in our study are in concordance with these data.

It is accepted that ECG changes after stroke may be associated with negative clinical implications. However, the role of ECG changes as predictors of neurologic outcome remains unknown. Importantly, the type of ST‐segment abnormality associated with poststroke prognosis also remains to be determined.

The main finding of our study was that ECG abnormalities are associated with a poor neurologic outcome 90 days after stroke. Specifically, both strain pattern and T‐wave alterations were predictors of neurologic disability. Another important issue is that these alterations were also predictors of mortality. Interestingly, both alterations suggest cardiac hypertrophy/ischemia and are associated with poor outcome in other clinical scenarios, including acute myocardial infarction, sudden death, and mainly in patients with hypertension.^28^ However, the role of strain pattern as predictor of poor neurologic outcome and mortality after stroke had not yet been demonstrated. Therefore, our data add prognostic information regarding these ECG changes in patients with stroke.

The clinical implications of our study are significant. The ability to obtain prognostic information upon a patient's admission to the emergency room by a fast, noninvasive, inexpensive method is of great value, especially where the timely institution of therapeutic measures is crucial, as in the case of stroke. Such information may, for example, influence decision‐making regarding thrombolysis in off‐label cases, whether or not a transient ischemic attack considered of low risk by other criteria or whether the patient would be better treated in a stroke unit or an intensive care unit. In short, we were able to identify a group of patients with stroke and a high risk of unfavorable evolution, thus allowing the early institution of specific monitoring as well as propaedeutic and therapeutic measures. In addition, strain pattern and T‐wave alterations in ECG at acute phase should involve various types of cardiovascular diseases. Therefore, we must emphasize the need of screening for cardiovascular disease, including acute coronary syndrome or several types of cardiomyopathy in this clinical scenario.

Another important issue is whether ECG abnormalities were acute or already preexisting at the time of brain injury. Unfortunately, we do not have prior ECGs from our patients. In a cohort such as ours, comprising many of the elderly, the high frequency of ECG abnormalities may be attributed to the frequency of ECG changes normally found in the elderly population. However, the frequencies of the different ECG changes present in the acute phase of stroke in this study are higher than those described in epidemiologic studies, both in national and international surveys. Indeed, in a national study of 1524 participants over 65 years of age in the city of São Paulo's urban population, the prevalence of AF was 2.4%, while the frequency of findings suggestive of ischemic heart disease was 12%.^29^ An analysis of four large European epidemiologic studies comprising 1680 participants over 64 years of age showed a prevalence of ST‐segment abnormalities as high as 9% and AF of 2.7%.^30^ Specifically for AF, which is a known risk factor for stroke,^31^ 15% of the patients included in the present study reported AF as a previous morbidity, similar frequency to that of 13% expected in our country.^32^ Interestingly, however, in the ECG performed at admission during the acute phase of stroke, the presence of such arrhythmias was 25%. Therefore, the discrepancy in the frequencies of ECG abnormalities found in this study and among the general population leads us to believe that acute neurologic injury plays a role in the genesis of some ECG alterations.

Several theories have already been formulated to explain changes in ECG after stroke. However, at this point, it is accepted that the ECG abnormalities are a manifestation of autonomic dysregulation caused by a lesion that affects areas representative of the autonomic nervous system, mainly the insula. In consequence, there would be a sympathetic overactivity. This, in turn, could induce cardiac toxicity, with a rapid development of subendocardial microinfarcts with calcification and the formation of contraction bands, called myocytolysis.^2^, ^3^, ^4^ This phenomenon can be detected by higher levels of biomarkers of myocardial necrosis, mainly troponin. Indeed, some studies have shown more ECG abnormalities in patients with stroke and high levels of troponin than in those with normal levels of troponin.^4^ However, importantly, we did not find an association between ECG abnormalities and biomarkers of myocardial necrosis and clinical stroke syndromes, suggesting that the pathophysiologic mechanisms involved in this scenario remain to be studied.

Finally, this study had some limitations. First, it included a relatively small sample of patients at a single hospital. In addition, we cannot know whether the ECG changes would have been seen prior to stroke or were acutely induced by neurologic injury. However, even in the case of ECG changes before stroke, they would not diminish the importance of the strain pattern and T‐wave abnormalities as predictors of poor prognosis in stroke patients treated in the emergency room. Therefore, despite these limitations, we strongly believe that our study adds important data about the role of ECG abnormalities as predictors of both mortality and poor neurologic outcome after stroke.

## 5 CONCLUSION

In conclusion, strain pattern and T‐wave alterations were predictors of both mortality and poor neurologic outcome 90 days after stroke. In addition, there was no association between ECG abnormalities with biomarkers of myocardial necrosis and clinical stroke syndromes.

## CONFLICT OF INTEREST

The authors declare no potential conflict of interest.

## References

[1] Hankey GJ . Stroke. Lancet. 2017;389:641‐654. 27637676 10.1016/S0140-6736(16)30962-X

[2] Tahsili‐Fahadan P , Geocadin RG . Heart‐brain axis: effects of neurologic injury on cardiovascular function. Circ Res. 2017;120:559‐572. 28154104 10.1161/CIRCRESAHA.116.308446

[3] Samuels MA . The brain‐heart connection. Circulation. 2007;116:77‐84. 17606855 10.1161/CIRCULATIONAHA.106.678995

[4] Manea MM , Comsa M , Minca A , Dragos D , Popa C . Brain‐heart axis‐review article. J Med Life. 2015;8:266‐271. 26351525 PMC4556904

[5] Zhang L , Qi S . Electrocardiographic abnormalities predict adverse clinical outcomes in patients with subarachnoid hemorrhage. J Stroke Cerebrovasc Dis. 2016;25:2653‐2659. 27476337 10.1016/j.jstrokecerebrovasdis.2016.07.011

[6] Coghlan LA , Hindman BJ , Bayman EO , et al. Independent associations between electrocardiographic abnormalities and outcomes in patients with aneurysmal subarachnoid hemorrhage: findings from the intraoperative hypothermia aneurysm surgery trial. Stroke. 2009;40:412‐418. 19095990 10.1161/STROKEAHA.108.528778 PMC2684783

[7] Christensen H , Boysen G , Christensen AF , Johannesen HH . Insular lesions, ECG abnormalities, and outcome in acute stroke. J Neurol Neurosurg Psychiatry. 2005;76:269‐771. 15654050 10.1136/jnnp.2004.037531 PMC1739524

[8] Tanaka M , Nakayama Y , Maeda Y , Nishioka T , Shirakawa M , Tsumura K . Electrocardiographic Q‐waves as a predictor of mortality in patients with cerebral infarction. Neurology. 2004;62:1818‐1821. 15159484 10.1212/01.wnl.0000125191.40444.8e

[9] Afsar N , Fak AS , Metzger JT , van Melle G , Kappenberger L , Bogousslavsky J . Acute stroke increases QT dispersion in patients without known cardiac diseases. Arch Neurol. 2003;60:346‐350. 12633145 10.1001/archneur.60.3.346

[10] Ibrahim GM , Macdonald RL . Electrocardiographic changes predict angiographic vasospasm after aneurysmal subarachnoid hemorrhage. Stroke. 2012;43:2102‐2107. 22678087 10.1161/STROKEAHA.112.658153

[11] Rahar KK , Pahadiya HR , Barupal KG , Mathur CP , Lakhotia M . The QT dispersion and QTc dispersion in patients presenting with acute neurological events and its impact on early prognosis. J Neurosci Rural Pract. 2016;7:61‐66. 26933346 10.4103/0976-3147.172173 PMC4750342

[12] McDermott MM , Lefevre F , Arron M , Foley J , Martin GJ , Biller J . Prognostic significance of ST‐segment depression on continuous electrocardiography in patients with acute ischemic neurologic events. J Stroke Cerebrovasc Dis. 1995;5:180‐184. 26486817 10.1016/S1052-3057(10)80172-7

[13] van Swieten JC , Koudstaal PJ , Visser MC , Schouten HJ , van Gijn J . Interobserver agreement for the assessment of handicap in stroke patients. Stroke. 1988;19:604‐607. 3363593 10.1161/01.str.19.5.604

[14] Wardlaw JM , Murray V , Berge E , del Zoppo GJ . Thrombolysis for acute ischaemic stroke In: Wardlaw JM, ed. Cochrane Database of Systematic Reviews. 2014:29:CD000213. 10.1002/14651858.CD000213.pub3 PMC4153726 25072528

[15] Gianni M , Dentali F , Grandi AM , Sumner G , Hiralal R , Lonn E . Apical ballooning syndrome or takotsubo cardiomyopathy: a systematic review. Eur Heart J. 2006;27:1523‐1529. 16720686 10.1093/eurheartj/ehl032

[16] Kumar S , Selim MH , Caplan LR . Medical complications after stroke. Lancet Neurol. 2010;9:105‐118. 20083041 10.1016/S1474-4422(09)70266-2

[17] Thygesen K , Alpert JS , Jaffe AS , et al. Third universal definition of myocardial infarction. Eur Heart J. 2012;33:2551‐2567. 22922414 10.1093/eurheartj/ehs184

[18] Myerburg R , Castellanos A . Cardiac arrest and sudden cardiac death In: Libby P, Braunwald E, eds. Organizadores. Braunwald's Heart Disease: a Textbook of Cardiovascular Medicine. Philadelphia, PA: Saunders/Elsevier; 2008:933.

[19] Calkins H , Kuck KH , Cappato R , et al. 2012 HRS/EHRA/ECAS expert consensus statement on catheter and surgical ablation of atrial fibrillation: recommendations for patient selection, procedural techniques, patient management and follow‐up, definitions, endpoints, and research trial design. Europace. 2012;14:528‐606. 22389422 10.1093/europace/eus027

[20] Yancy CW , Jessup M , Bozkurt B , et al. 2013 ACCF/AHA guideline for the management of heart failure. J Am Coll Cardiol. 2013;62:e147‐e239. 23747642 10.1016/j.jacc.2013.05.019

[21] Goldhaber SZ , Bounameaux H . Pulmonary embolism and deep vein thrombosis. Lancet. 2012;379:1835‐1846. 22494827 10.1016/S0140-6736(11)61904-1

[22] Martins SCO , de Freitas GR , Pontes‐Neto OM , et al. Guidelines for acute ischemic stroke treatment: part II: stroke treatment. Arq Neuropsiquiatr. 2012;70:885‐893. 23175203 10.1590/s0004-282x2012001100012

[23] Horan TC , Andrus M , Dudeck MA . CDC/NHSN surveillance definition of health care‐associated infection and criteria for specific types of infections in the acute care setting. Am J Infect Control. 2008;36:309‐332. 18538699 10.1016/j.ajic.2008.03.002

[24] Davenport RJ , Dennis MS , Warlow CP . Gastrointestinal hemorrhage after acute stroke. Stroke. 1996;27:421‐424. 8610306 10.1161/01.str.27.3.421

[25] Mehta RL , Kellum JA , Shah SV , et al. Acute kidney injury network: report of an initiative to improve outcomes in acute kidney injury. Crit Care. 2007;11:R31. 17331245 10.1186/cc5713 PMC2206446

[26] Kassim T , Clarke D , Mai V , Clyde P , Mohamed Shakir K . Catecholamine‐induced cardiomyopathy. Endocr Pract. 2008;14:1137‐1149. 19158054 10.4158/EP.14.9.1137

[27] Fye WB . A history of the origin, evolution, and impact of electrocardiography. Am J Cardiol. 1994;73:937‐949. 8184849 10.1016/0002-9149(94)90135-x

[28] Okin PM , Devereux RB , Nieminen MS , et al. Electrocardiographic strain pattern and prediction of cardiovascular morbidity and mortality in hypertensive patients. Hypertension. 2004;44:48‐54. 15173125 10.1161/01.HYP.0000132556.91792.6a

[29] Kawabata‐Yoshihara LA , Benseñor IM , Kawabata VS , Menezes PR , Scazufca M , Lotufo PA . Prevalence of electrocardiographic findings in elderly individuals: the Sao Paulo aging & health study. Arq Bras Cardiol. 2009;93:602‐607. 20379640 10.1590/s0066-782x2009001200015

[30] de Bacquer D , De Backer G , Kornitzer M . Prevalences of ECG findings in large population based samples of men and women. Heart. 2000;84:625‐633. 11083741 10.1136/heart.84.6.625 PMC1729526

[31] Wolf PA , Abbott RD , Kannel WB . Atrial fibrillation as an independent risk factor for stroke: the Framingham study. Stroke. 1991;22:983‐988. 1866765 10.1161/01.str.22.8.983

[32] O'Donnell MJ , Xavier D , Liu L , et al. Risk factors for ischaemic and intracerebral haemorrhagic stroke in 22 countries (the INTERSTROKE study): a case‐control study. Lancet. 2010;376:112‐123. 20561675 10.1016/S0140-6736(10)60834-3

